# MOF-Derived AlCuSe_2_ Embedded in a Carbon Matrix for an Economical Anode of Lithium-Ion Battery

**DOI:** 10.1021/acsomega.2c03819

**Published:** 2022-08-22

**Authors:** Muhammad Ali, Muhammad Tayyab Ahsan, Ahtisam Mehmood, Ayesha Ishfaq, Ghulam Ali, Muhammad Aftab Akram, Sofia Javed, Zeeshan Ali

**Affiliations:** ^†^School of Chemical and Materials Engineering (SCME), ^‡^School of Interdisciplinary Engineering & Sciences, ^§^U.S.-Pakistan Center for Advanced Studies in Energy (USPCAS-E), National University of Sciences and Technology (NUST), H-12, Islamabad 44000, Pakistan; ∥ School of Materials Science and Engineering, Peking University, Beijing 100871, China; ⊥ Department of Materials Science & Engineering, Pak-Austria Fachhochschule, Institute of Applied Sciences & Technology, Khanpur Road, Mang, Haripur 22650, Pakistan

## Abstract

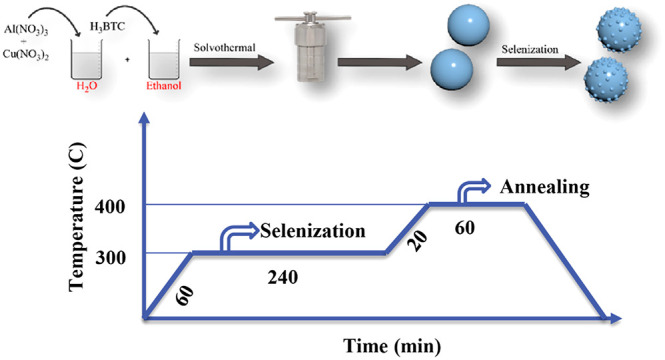

Binary metal chalcogenides (TMCs) have emerged as a potential candidate for lithium-ion batteries due to their availability, abundance, chemical properties, and high theoretical capacities. Despite these characteristics, they suffer from significant volume change, limited life cycle, and inferior rate capabilities which hinder their practical applications. These issues can be addressed by selecting low-cost nanostructure metal combinations coupled with a carbon matrix, which tackles significant volume change to give prolonged cycle life and high-rate capabilities. Herein, novel MOF-derived aluminum copper selenide (ACSe@C) nanospheres embedded in a carbon matrix are synthesized via a facile solvothermal route. Owing to their uniform porous structure, ACSe@C nanospheres exhibit excellent electrochemical performance as an anode material for Li-ion batteries. ACSe@C delivers a high specific capacity of 633.6 mAh g^–1^ at 0.1 A g^–1^ and a good rate capability of 532 mAh g^–1^ at 0.1 A g^–1^ and 400 mAh g^–1^ at 8 A g^–1^. This study demonstrates that ACSe@C is a good candidate for next-generation energy-storage devices.

## Introduction

Environmental issues, such as global warming and climate change brought about by fossil fuels, negatively influence all global economies.^1,2^ To meet the rapid growth in energy demand while minimizing environmental concerns, it is critical to producing renewable energy. However, most renewable sources, such as wind, solar, and tidal, are infrequent, demanding highly efficient, long-life, and low-cost energy storage systems.^3,4^ To achieve these goals, novel battery technology and advanced material development are required immediately, where batteries must have high electrochemical performance and good cycle life. When this type of situation occurs, it is very demanding to develop batteries that have high performance and can fulfill a variety of needs, which almost certainly requires additional advancements in battery materials.^5−9^ Globally, there has been a surge of interest in developing electrochemical power devices such as batteries.^10^ In the last century, nonaqueous rechargeable Li-ion batteries have been one of the most effective energy storage systems in modern materials electrochemistry.^11−14^ Low-voltage aquas batteries such as Ni–Cd^15^ and Ni–MH^16^ systems have gradually been replaced by Li-ion technology since 1991, when Sony Corporation launched the first Li-ion battery for small electronic devices. Li-ion batteries are widely employed in portable devices nowadays, including robots, various power tools, stationary power storage units, and electric cars.^17^ However, traditional Li-ion battery technologies fall short of meeting these requirements.^12,18^ Graphite is undeniably a good choice for the anode in Li-ion batteries. Due to its superior properties, graphite is extensively used as an anode material in commercial Li-ion batteries. These properties include low working potential, low cost, and long cycle life.^19^ The main problem with graphite is that it has a low capacity of 372 mAh g^–1^. Due to intercalation, there is only one Li^+^ for every six carbon atoms, which results in a stoichiometric LiC_6_. Batteries with a graphite anode typically have a poor power density owing to the slow diffusion of Li^+^ through the carbon layers.^20^ As a result, it is critical to develop novel anode materials with increased capacity and lithium-ion diffusion rates to increase energy and power densities.

High capacity metals (P,^21^ Sn,^22^ Sb^23^) and metal compounds (oxides,^24^ sulfides,^25^ phosphides,^26^ and selenides^27,28^) are some of the new anode materials that have been prepared. Among these, binary metal chalcogenides (BMCs) as anodes for LIBs have been the scientific community’s focus in recent years. The theoretical capacity of BMCs is substantially greater. Other advantages of BMCs are their low cost, abundance, and ease of fabrication; these features are highly useful for their use in electrical devices. More critically, these properties make BMCs potential electrode candidates for suggested electrochemical energy storage devices. Although they have many advantages, they have disadvantages as well. Due to high pulverization in binary metal chalcogenide electrodes, they suffer from poor cycling stability during the (dis)charging process, which results in fading capacity.^24,29−31^

Binary metal chalcogenides have made promising progress as the anode materials for energy storage systems due to their extraordinary properties. It has been reported that binary metal chalcogenides have excellent electrical conductivity due to their comparatively narrow band gap and low activation energy for electron transport between cations, resulting in improved electrochemical characteristics.^32^ Binary metal chalcogenides give better performance than monometal chalcogenides.^33^ In general, binary metal chalcogenides have considerable structural and chemical benefits over monometallic chalcogenides, increasing their electrochemical properties. The existence of multiphases in binary metal chalcogenides results in numerous phase boundaries and tiny crystalline domains, which aids in bypassing solid-state diffusion and enabling fast ionic diffusion kinetics.^34^ There are more reduction and oxidation sites in binary metal chalcogenides due to the multiple valences of cations present in them. Hence, there is a higher chance of electron reactivity for these compared to monometal chalcogenides, which ultimately leads to higher electrochemical activity. For example, Mn/Fe MOF was used as template for the synthesis of biphasic Fe_7_S_8_@MnS encapsulated in a carbon matrix for anode material in a Li-ion battery it delivers 581 mA h g^–1^ after 500 cycles at 1 A g^–1^ and exhibits good reversible capacity.^35^ Jin and co-workers studied binary metal selenides (ZnSe/CoSe) which are encapsulated in a nitrogen-doped carbon matrix interconnected with carbon nanotubes. They achieve a high stable capacity of 786 mAh g^–1^ after 1000 cycles at the current density of 1 A g^–1^.^36^

For the first time, we have used a simple hydrothermal technique followed by a selenization process to produce low-cost hierarchically nano porous spheres of aluminum copper selenide (AlCuSe_2_, termed as ACSe@C). ACSe@C shows uniform spherical morphology and a high surface area of 97.83 m^2^g^–1^. ACSe@C shows strong oxidation and reduction peaks in cyclic voltametery. It also shows a good initial discharge capacity of 455.5 mA h g^–1^ at 4 A g^–1^. ACSe shows a good capacity retention of 71% even after 2000 cycles at a current density of 4 Ag^–1^.

## Results and Discussion

Herein, for the first time, we have synthesized hierarchically porous spheres of aluminum copper binary-metal selenide (AlCuSe_2_, termed as ACSe@C) using a facile hydrothermal process followed by selenization and an annealing strategy.

**Figure 1:**
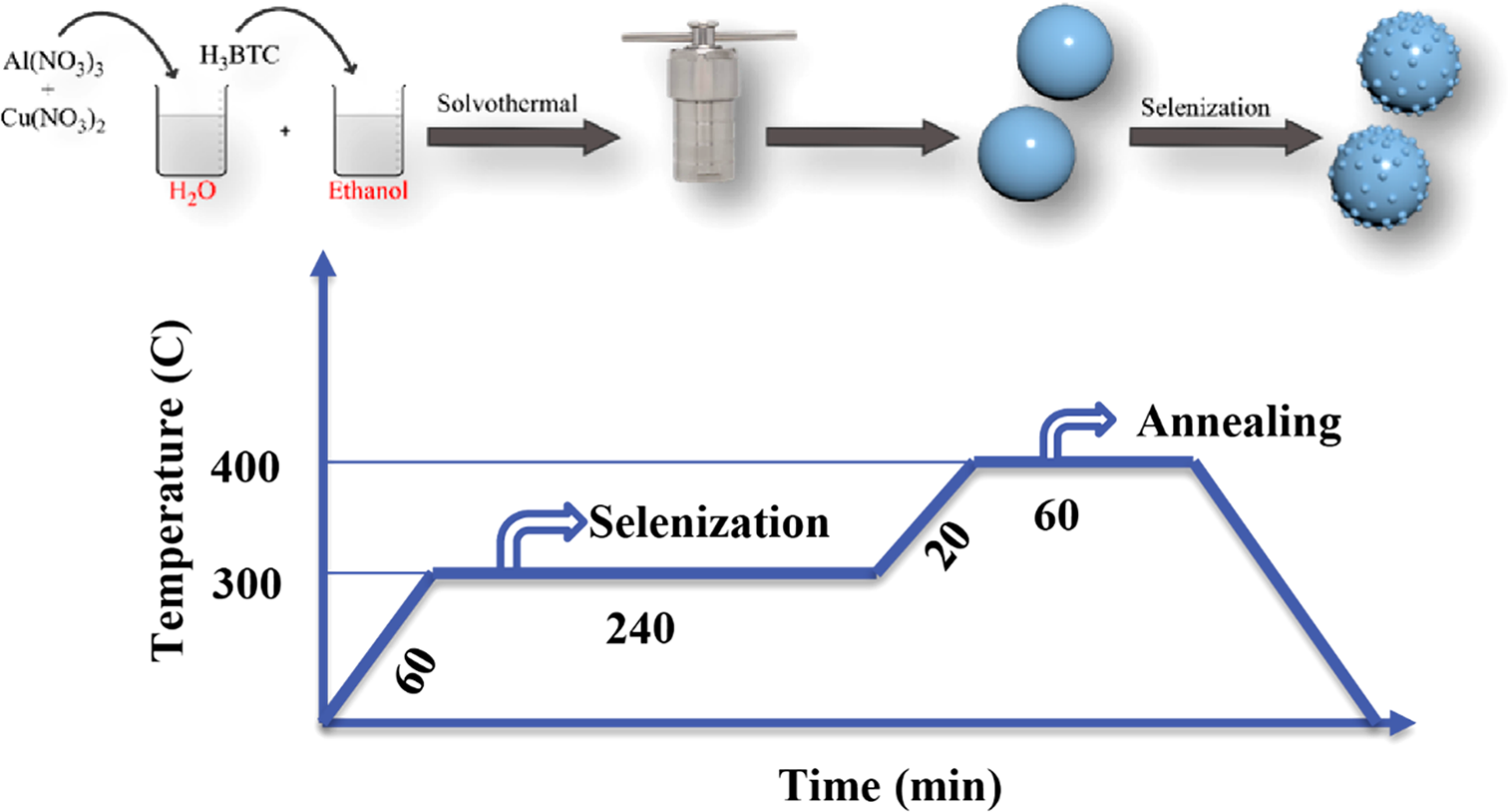
Synthesis and selenization conditions for ACSe@C.

**Figure 2:**
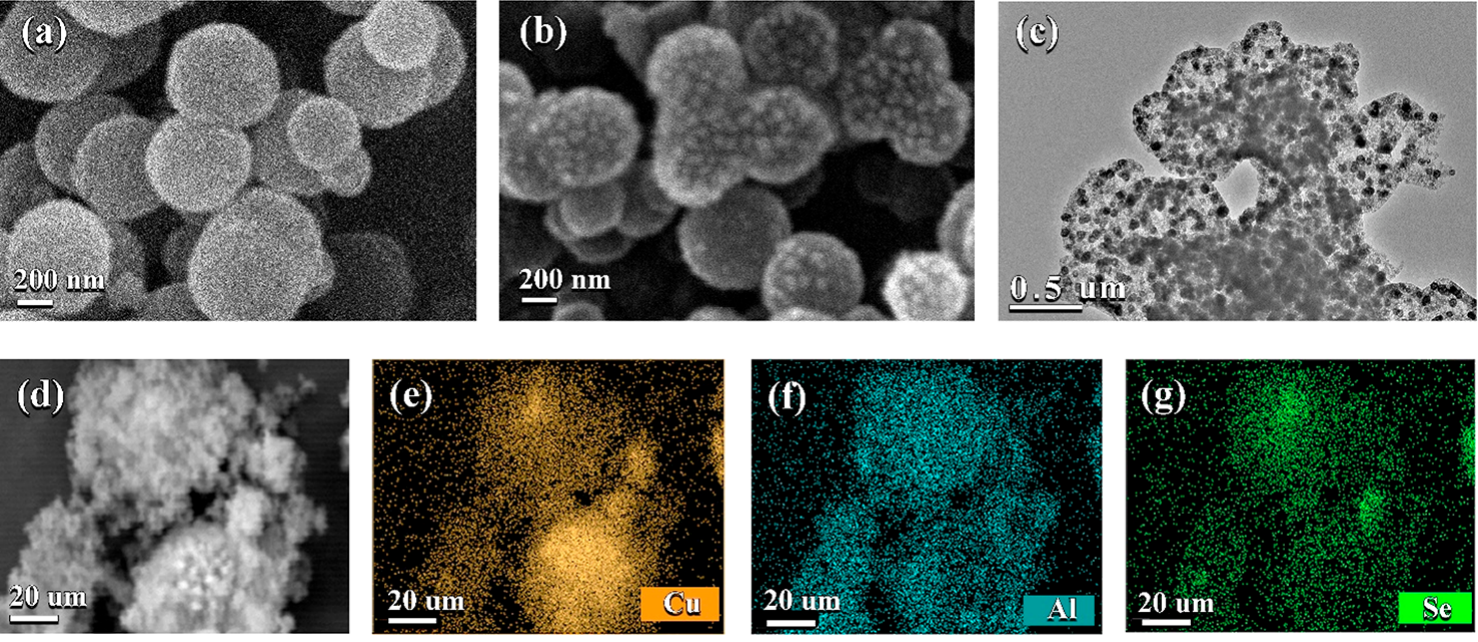
(a) SEM of Al–Cu MOF, (b) SEM of ACSe@C, (c) TEM of ACSe@C, (d–g) elemental mapping of ACSe@C.

Figure 1 shows a schematic illustration of the formation process of AlCuSe_2_ (ACSe@C). Hydrothermal treatment results in Al–Cu MOF precursor spheres, and combined selenization and annealing produce hierarchically porous ACSe@C spheres made of nanoparticles. The morphology of the prepared ACSe@C) was characterized by using (SEM, TEM) as shown in Figure 2a,b. Al–Cu MOF with uniform spherical morphology is shown in Figure 2a. The average nanosphere size is around 350 nm. Figure 2b represents the ACSe@C after selenization. ACSe@C nanoparticles are embedded in the carbon matrix. This carbon matrix compensates for the volume changes during the lithiation and delithiation processes and enhances the electronic conductivity. Figure 2c shows that ACSe@C embedded nanoparticles in the carbon matrix were successfully synthesized. The figure clearly shows the stable nanograin formation in the background of the carbon structure with very high achieved porosity. These properties are essential for the electrochemical performance of the anode material of Li-ion batteries. The volume change problem of the electrode material during the lithiation and delithiation processes is tackled by the carbon matrix that lowers the diffusion length and results in a minimum volume change. The carbon structure acts as a bed to limit the volume changes of the material and possible material agglomeration throughout the charging and discharging process. Based on this analysis, it was confirmed that the binary metal selenide nanoparticles embedded in the carbon matrix were successfully synthesized. The distribution of elements such as Al, Cu, and Se in ACSe@C was analyzed through EDX elemental mapping analysis in scanning electron microscopy. Figure 2d–g shows SEM images of copper selenide, and aluminum selenide is shown in Figures S5 and S6. EDX maps show the uniform distribution of all elements. Moreover, there is no stray Se in the mapping, which shows the complete selenization of Al–Cu MOF.

**Figure 3:**
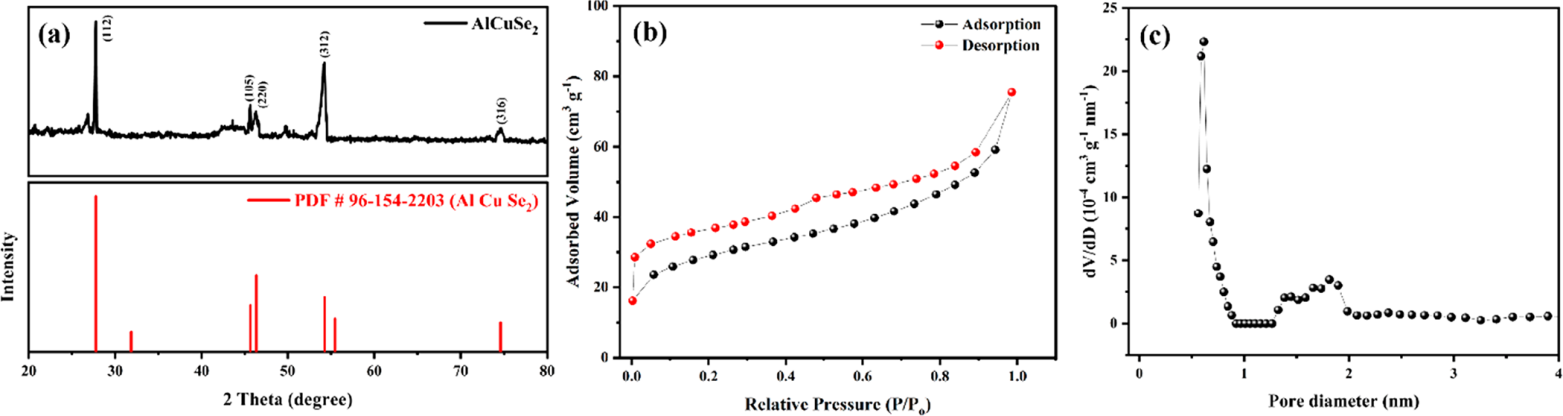
(a) XRD graph of ACSe@C. (b) N_2_ adsorption–desorption isotherms of ACSe@C. (c) Corresponding pore size distribution.

XRD was carried out to study the crystal structure and compositional analysis of synthesized ACSe@C Figure 3a. The XRD pattern endorsed the formation of a homogeneous tetragonal structure of AlCuSe_2_, which is a preliminary agreement with PDF Card No. 96-154-2203. There is no peak of impurity observed in the XRD pattern. The maximum X-diffraction was observed along the crystal plan (112) assigned at the diffraction position of 2θ = 27.750°. The other peaks observed as 45.617°, 46.262°, 54.276°, and 74.694° correspond to the plans (101), (220), (312), and (316), respectively. XRD of CuSe_2_ and Al_2_Se_3_ are also shown in Figures S3 and S4. The pore size and surface area are analyzed by the Brunauer–Emmett–Teller (BET) method. The isotherms shown in Figure 3b can be categorized as types IV and II. These types of graphs show the presence of micro and mesopores simultaneously giving the obtained material hierarchically by nature of porosity.^37^ the ACSe@C nanosphere exhibits a high surface area of 97.83 m^2^ g^–1^. It is revealed that the material has micropores of width 0.6 nm (Figure 3c). The presence of micro- and mesopores and high surface area not only enhance the Li^+^ ion surface interaction but also increase the electronic conductivity by shortening the ion diffusion length; hence, these factors play an essential role in improving the electrochemical properties of the material.^38^

**Figure 4:**
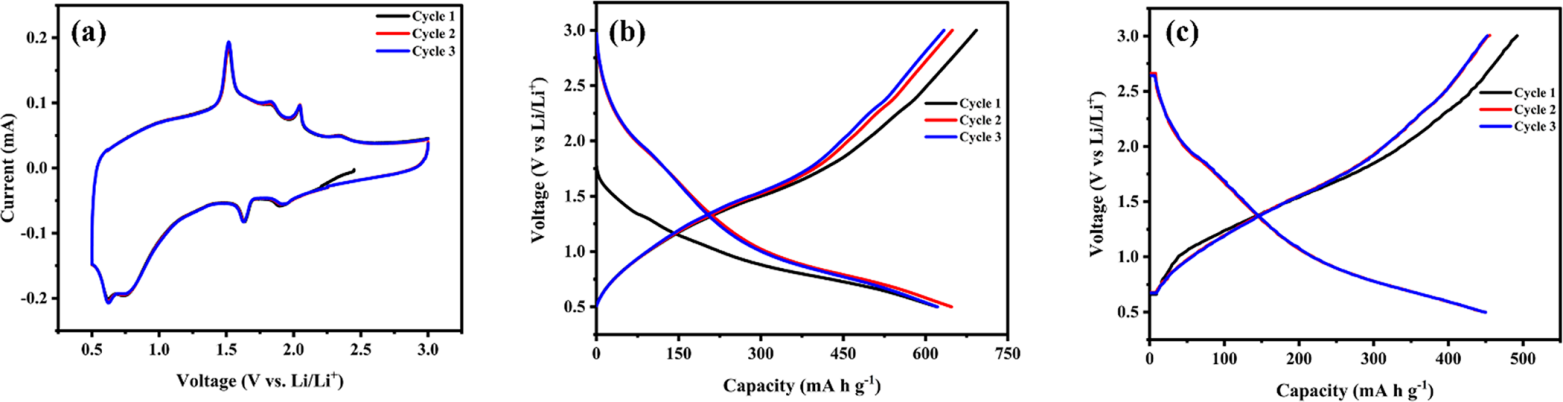
(a) CV curves of the ACSe@C anode in lithium half-cells of first three cycles at rate of 0.2 mV S^1–1^. (b) Electrochemical discharge and charge profiles (between 0.5 and 3.0 V) of the ACSe anode at a current rate of (b) 0.1 A g^–1^ and (c) 4 A g^–1^

The electrochemical properties of ACSe@C nano spheres were further evaluated by assembling the Li-half cell. Cyclic voltammetry (CV) curves of the first three cycles at 0.2 mV s^–1^are shown in Figure 4 a. CV curves of ACSe reveal various reduction and oxidation peaks in the voltage range of 0.5–3 V. The first peak is observed at ∼0.62 V and can be associated with Li_2_Se. The following cycles show more redox peaks at voltages of ∼0.75, ∼1.63, and ∼1.91 V. These peaks can be attributed to the transformation of metal selenides to Li_2_Se due to the insertion of Li^+^ ions. The cycles show good overlapping, and the area under each cycle was almost the same, which can be ascribed to reversible and stable cycling performance for anode material. Figure 4b,c shows the charge–discharge profile of the first three cycles of ACSe@C at 0.1 and 4 A g^–^^1^. In Figure 4b, ACSe@C shows a high initial discharge capacity of 692.6 mA h g^1^ between the voltage window of 0.5–3 V at 0.1 A g^–^^1^. The following three cycles show overlap with the capacity retention of 693.6 mA h g^–1^. The capacity loss between the first and succeeding cycles is ∼8.51%.

Figure 4c shows the high initial discharge capacity of 455.5 mA h g^–1^ of ACSe@C under a voltage window of 0.5–3 V at a high scan rate of 4 A g^–^^1^. The subsequent cycles show overlap with the capacity retention of 447.7 mA h g^–1^. The capacity loss between the first cycle and the succeeding cycles is ∼8–10%. All the observed plateaus in charge/discharge satisfy CV results. The overall good performance of the ACSe@C is due to the larger amount of nano porous carbon which leads to the greater surface area and porosity that result in the high lithium storage capacity and better rate capability.

**Figure 5:**
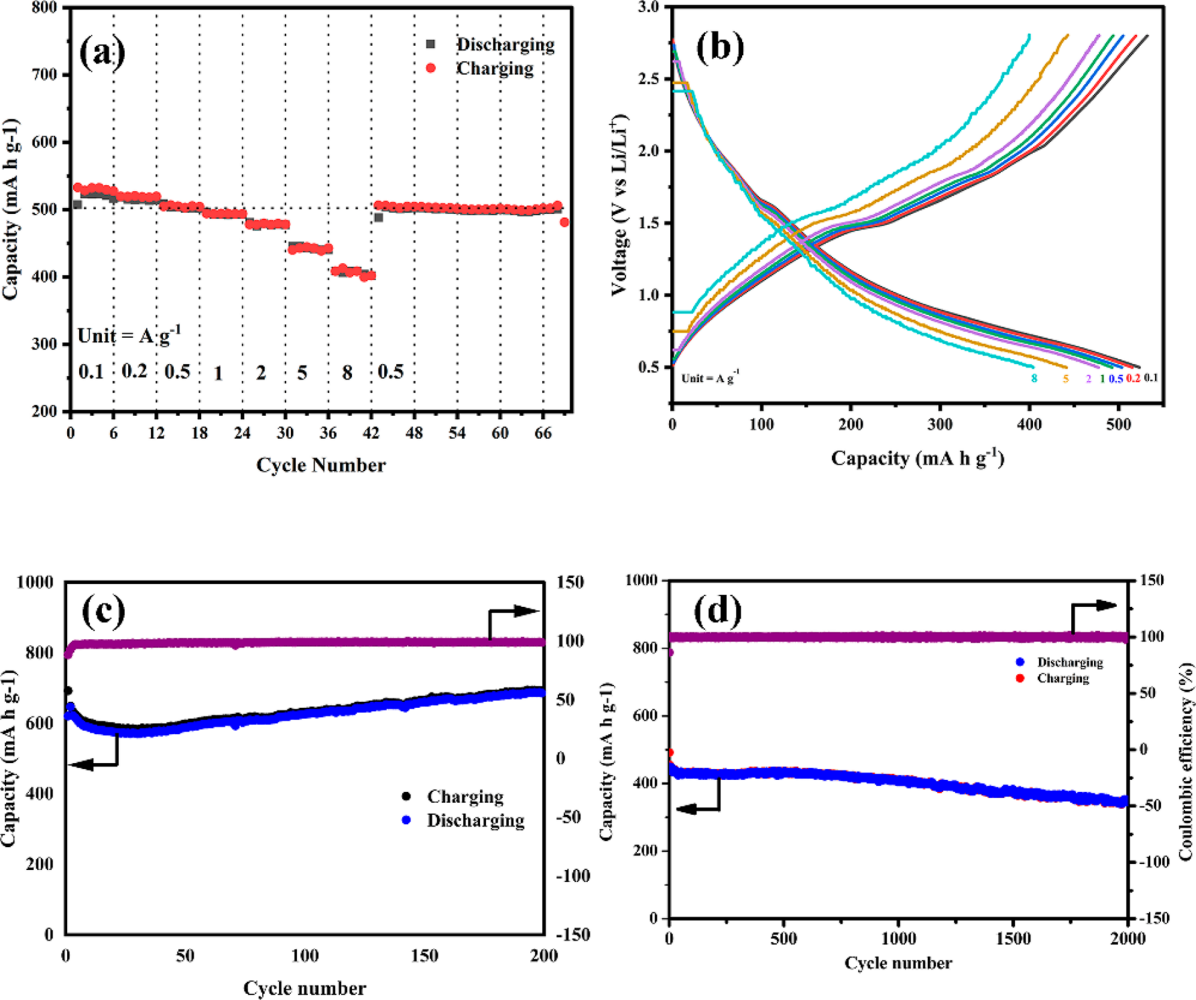
(a) Rate performances of ACSe@C at various current densities ranging from 0.1 to 8 A g^–1^. (b) Charge/discharge profiles at corresponding current densities. (c, d) Cycling performances of ACSe@C at a current density of (c) 0.1 A g^–1^ and (d) 4 A g^–1^.

The capacity retention of ACSe@C was evaluated by charging and discharging at different current rates as shown in Figure 5a. At high current density, anode material shows excellent performance. ACSe@C can deliver 401.9 and 442.9 mA h g^–1^ even at high current rates of 5 and 8A g^–1^, respectively. When current densities were increased from 0.1 to 0.2, 0.5, 1, 2, 5, and 8 A g^–1^ the specific capacities were 572.2, 519.5, 504.1, 493.8, 478.0, 442.9, and 401.9 mA h g^–1^, respectively, and when current rate varied reversely to 0.5 A g^–1^ then the specific capacity was fully recovered to 505.9 mA h g^–1^ indicating the excellent rate capability and reversibility of ACSe@C. Figure 5b shows the charge/discharge profile of increasing current densities. This also shows that electrode fully recovered specific capacity at 0.5 A g^–1^. This is due to their porous structure which allows more electrolyte to penetrate the electrode, and the carbon matrix acts as a bed which compensates for the volume change, hence resulting in high-rate capability. The long cyclic performance of ACSe@C at 0.1 and 4 A g^–1^ is shown in Figure 5c,d. ACSe@C shows a stable and reversible discharge capacity of 692.8 mA h g^–1^ with a capacity retention of 100% after 200 cycles at a current density of 0.1 A g^–1^. Even at a high current density of 4 A g^–1^ with a capacity retention of 71% for 2000 cycles it exhibits a high specific capacity of 351 mA h g^–1^. When compared with the reported literature (Table S1), we can conclude that the performance of ACSe@C under the same conditions is significantly better owing to the synergistic effect of binary metal combination and carbon embedment. The initial Coulombic efficiency of LIB anode materials is a key parameter for the improvement of energy density in batteries. Coulombic efficiency always stayed between 101.48% and 99.11%

## Conclusion

In summary, MOF-derived AlCuSe_2_ was successfully synthesized via a solvothermal approach. The unique structure in which ACSe@C was embedded into the carbon matrix helps to achieve the stabilized lithium insertion and extraction during the charging and discharging process. The excellent specific capacity of 692.8 mA h g^–1^ after 200 cycles with a capacity retention of 100%, reversibility, and rate capability were achieved during the charging and discharging process as anode material of the Li-ion battery. In attaining the desired electrochemical performance, uniformity was achieved, and the structural integrity of the nano porous framework entertained the central part to enhance its performance. The high surface area, morphology, and high porosity that lead to proper accessibility of the lithium ion toward active sites play a crucial role in achieving reversibility and high-rate capability. This research gives the potential to develop economical, stable, and better anode materials for Li-ion batteries and can lead to large-scale applications with high energy storage demands.

## Experimental Section

### Synthesis of Copper–Aluminum MOF

All reagents used for synthesis were of analytical grade purity and used in as-received form without further modification.

Copper–aluminum MOF was synthesized by the solvothermal method. Al(NO_3_)_3_·9H_2_O (1.876 g, 5 mmol) and Cu(NO_3_)_2_·3H_2_O (1.208 g, 5 mmol) were dissolved in 30 mL of deionized water (solution A). Benzene-1,3,5-tricarboxylic acid (1.261 g, 3 mmol) was dissolved in 30 mL of ethanol (solution B). Solution A was mixed with solution B and stirred for 10 min. Next, the solution was poured into a 100 mL Teflon-lined autoclave and maintained at 120 °C for 24 h. Particles were collected and rinsed in ethanol and deionized water through centrifugation. Afterward, particles were dried in vacuum oven at 100 °C for 12 h; similarly, for comparison aluminum and copper MOF were also synthesized as shown in Figures S1 and S2.

### Synthesis of Aluminum Copper Selenide

Al–Cu Se was obtained by a gas selenization process. Al–Cu MOF precursor and well-ground selenium powder (in weight ratio of 1:2) were placed at the opposite ends of the alumina boat. With increasing temperature (to 400 °C), selenium powder melts and reacts with incoming H_2_ gas to form H_2_Se locally (i.e., H_2_ + Se → H_2_Se). H_2_Se is a strong reducing agent, so it swiftly reduces the Al–Cu MOF precursor and converts it into ACSe@C. The boat was covered with Al-foil, and selenization was carried out at 300 °C for 4 h in a tube furnace with a ramping rate of 5 °C/min under the Ar/H_2_ (10%vol H_2_) environment. Then annealing was carried out at 400 °C for 1 h with a ramping rate of 5 °C/min. Finally, the selenized product was cooled naturally to room temperature.

### Material Characterization

An FEI Tecnai F30 (transmission electron microscope, TEM) and a scanning electron microscope (JEOL JSM6490A) with an EDX Z2-i7 analyzer detector attached to the SEM were used for the morphological characterization of the product. Brunauer–Emmett–Teller multipoint technology was used to calculate surface areas, pores, and pores (Quantachrome NovaWin 20e instrument, Virginia). Electrochemical characteristics were investigated using a LAND battery tester and workstation CHI760C.

### Electrochemical Characterization

The working electrode was prepared by mixing active material (ACSe@C) (70 wt %), carbon black (15 wt %), and carboxymethylcellulose sodium (15 wt %) in DI water. A homogenous slurry was pasted on well cleaned Cu foil and dried at 70 °C overnight in a vacuum oven. To prepare the lithium half-cell, 2023-type coin cells were fabricated in an Ar-filled glovebox. Prepared electrodes were cut into small pieces of diameter 12 mm, and circular lithium disks of diameter 14 mm were used for the reference and counter electrode, a micro glass fiber of diameter 16 mm used as the separator, and 1 M LiPF_6_ in a 1:1 in a mixture of ethylene carbonate and diethylene carbonate was used as the electrolyte. The mass of electrode was found to be 0.4500 mg.

## Supporting Information Available

Synthesis of copper MOF (Figure S1); synthesis of aluminum MOF (Figure S2); XRD analysis of copper selenide (Figure S3); XRD analysis of aluminum selenide (Figure S4); SEM analysis of Cu_2_Se (Figure S5); SEM analysis of Al_2_Se_3_ (Figure S6) (PDF)
