# Supplementary material for: MOF-Derived AlCuSe2 Embedded in a Carbon Matrix for an Economical Anode of Lithium-Ion Battery
Source: ACS Omega. 2022 Aug 22;7(34):30440–6. doi: 10.1021/acsomega.2c03819 (PMC9434617; doi:10.1021/acsomega.2c03819)
Supplement: Supplementary file 1 — ao2c03819_si_001.pdf [file ao2c03819_si_001.pdf]

Supporting Information

MOF Derived $AlCuSe_2$ embedded on carbon matrix for an economical anode of Lithium-ion battery

Muhammad Ali[1,2], Muhammad Tayyab Ahsan[1,4], Ahtisam Mehmood[1], Ayesha Ishfaq[1], Ghulam Ali[3], Muhammad Aftab Akram[1,5], Sofia Javed[1], Zeeshan Ali[1,2,*]

Affiliations

[1]School of Chemical and Materials Engineering (SCME), [2]School of Interdisciplinary Engineering & Sciences, [3]USPCAS-E, National University of Sciences and Technology (NUST), H-12, Islamabad, 44000 Pakistan

[4]School of Materials Science and Engineering, Peking University, Beijing, 100871, China.

[5]Department of Materials Science & Engineering, Pak-Austria Fachhochschule, Institute of Applied Sciences & Technology, Khanpur Road, Mang, Haripur 22650, Pakistan

Email: zeeshan.ali@scme.nust.edu.pk

Synthesis of Copper MOF

Copper MOF was synthesized by solvothermal method a schematic route is shown in fig copper nitrate trihydrate (5 g, 20 mmol) and 1,3,5-Benzene tricarboxylic acid (2.5 g, 12mmol) were sonicated for 15 min in a solution containing an equal amount of Di-methyl-formamide (DMF), Ethanol and DI water in a beaker. Then this solution was placed in a heating oven at 75°C for 24 hours. After those particles were collected through centrifugation at Rpm of 4000/min. In last particles were washed with DMF 3 times to remove the unreacted species.

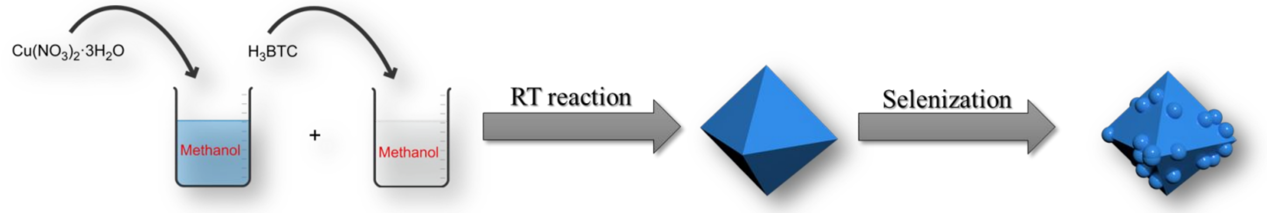

Figure S1 Synthesis of Copper MOF

Synthesis of Aluminum MOF

Aluminum MOF was synthesized by the solvothermal method. Aluminium nitrate nonahydrate (1.12 g, 3 mmol) was dissolved in 30ml of Deionized water (solution A). 1,3,5-Benzene tricarboxylic acid (0.63 g, 3 mmol) was dissolved in 30ml of Dimethylformamide (DMF) (Solution B). After that Solution B was mixed with Solution A and stirred for 10 min. This solution was transferred into a 100ml Teflon-lined autoclave and kept at 120°C for 10 hours. Particles were collected through centrifugation at 4000 rpm. After that washed with Dimethylformamide (DMF) and Deionized water 3 times. Particles were dried at 100 for 12hr in a vacuum oven.

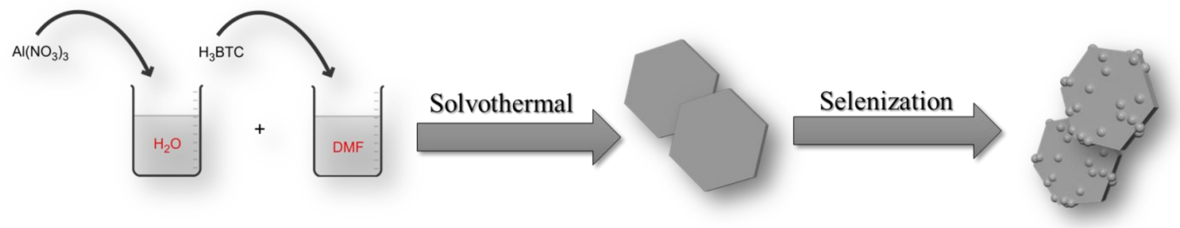

Figure S2 Synthesis of Aluminum MOF

XRD analysis of copper selenide

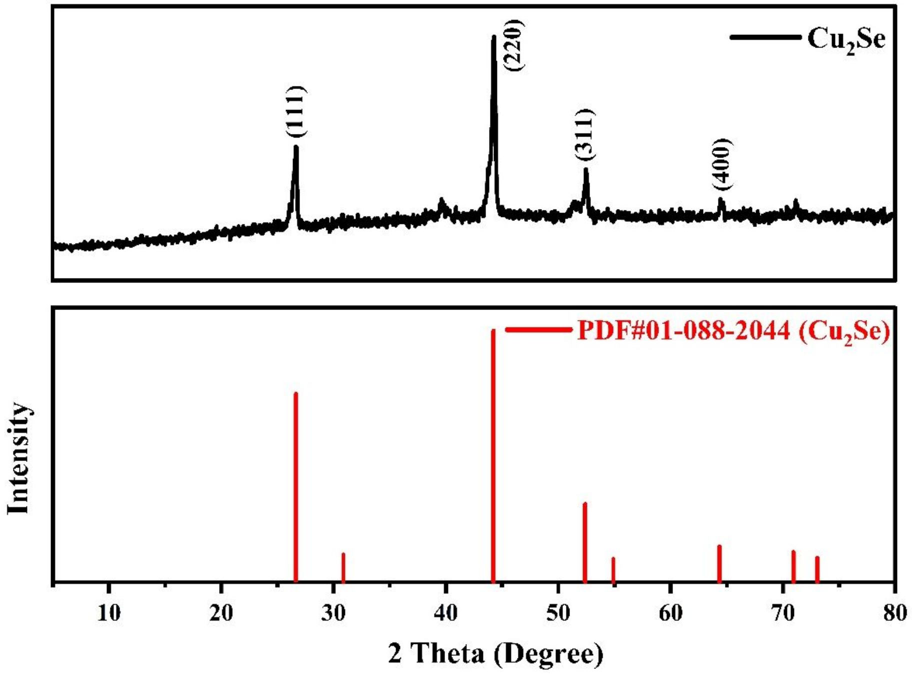

Figure S3 XRD analysis of Cu_2Se

XRD was carried out to study synthesized Copper selenide's structural and compositional analysis. The obtained XRD pattern endorsed the formation of a homogeneous cubic structure of Cu2Se, which is a preliminary agreement with PDF Card # 01-088-2044. There is no peak of impurity observed in the XRD pattern. The maximum X-ray-diffraction was observed along the crystal plan (220) assigned at a diffraction position of $2\theta = 44.234^{O}$. It shows that in this plan, synthesized material is more stable. The other peaks observed as 26.660^{O}, 52.396^{O}, 64.342^{O} corresponds to the plans (111), (311), and (400), respectively.

XRD analysis of Aluminum selenide

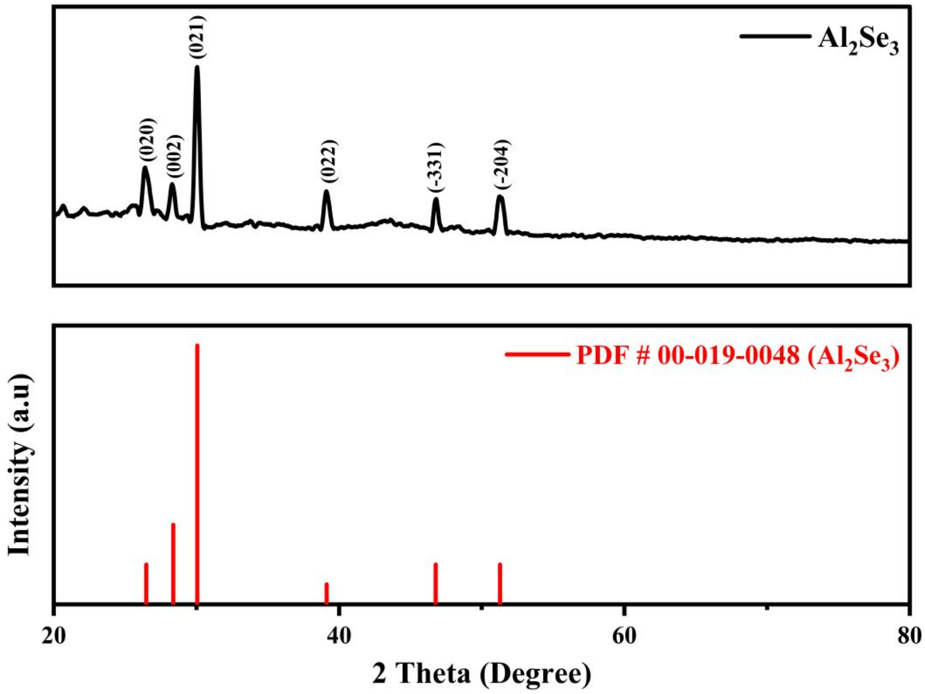

Figure S4 XRD analysis of Al_2Se_3

XRD was carried out to study the structural and compositional analysis of synthesized Aluminum selenide. The obtained XRD pattern endorse the formation of a homogeneous Monoclinic structure of Al_2Se_3 which is in complete agreement with PDF Card # 00-019-0048. There is no peak of impurity was observed in the XRD pattern. The maximum X ray-diffraction was observed along the crystal plan (021) assigned at a diffraction position of $2\theta = 30.041^{O}$. It shows that in this plan, synthesized material is more stable. The other peaks observed as 26.240 ^{O}, 28.249 ^{O}, 38.979 ^{O}, 46.760 ^{O}, 51.092 ^{O} corresponds to the plans (020), (002), (022), (-331), and (-204) respectively.

SEM analysis of Cu_2Se

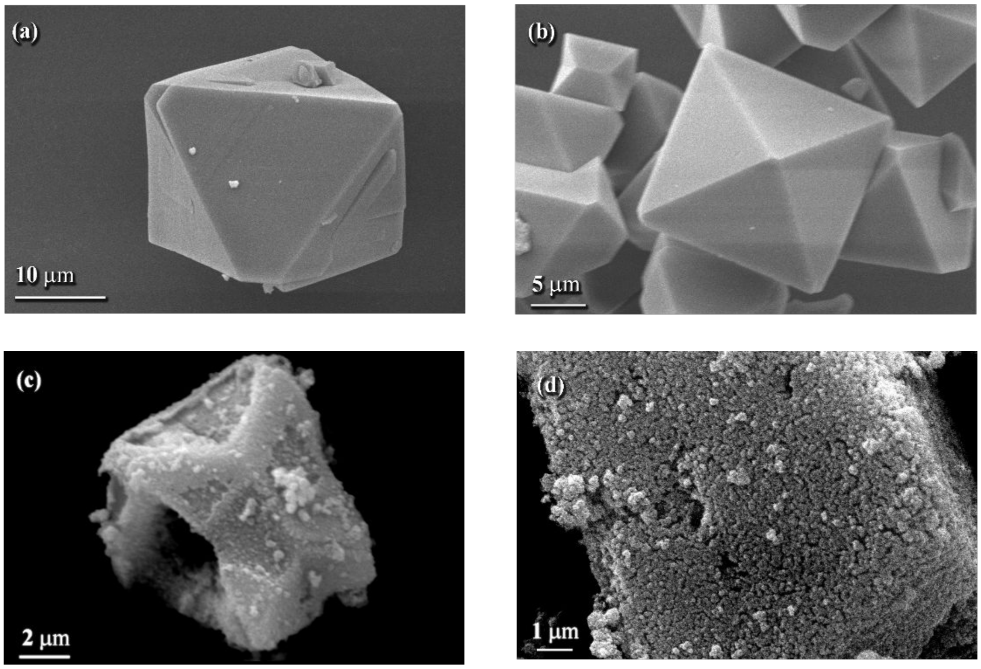

Figure S5 (a-b) Cu-MOF (c-d) Cu-Se

Figure (a-b) shows the Copper MOF. From SEM images, it is revealed that Copper MOF has uniform morphology. Figure (c-d) shows the Copper selenide. It is revealed that morphology is almost retained. The carbon matrix encapsulated copper selenide nanoparticles.

SEM analysis of Al_2Se_3

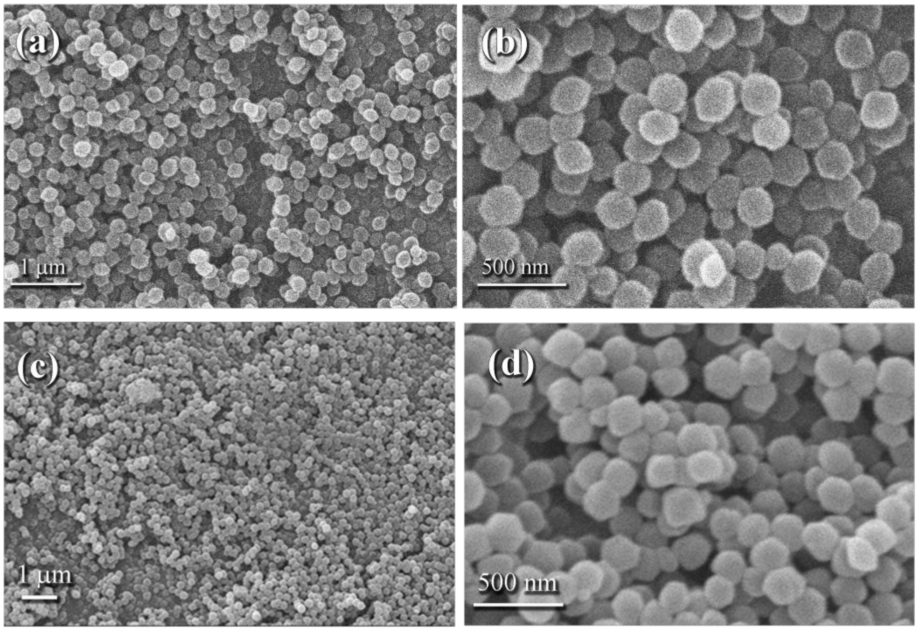

Figure S6 (a-b) SEM of Al MOF (c-d) SEM of Al-Se

Figure (a-b) shows the Al-MOF. From the images, uniform hexagonal morphology is obtained. Figure (c-d) shows the Al-Se it is also clear that after the selenization, morphology remains intact, and Al-Se is successfully synthesized.

Table S1: Comparison of lithium-ion storage performance of our work with already reported literature.

**Materials**	**Morphology**	**Performance capacity (mAh g^{-1}) rate (A g^{-1}) cycle number**	**Reference**
$FeSe_2$@C	Spherical	474.7/1/100	1
$NiSe_2$@C	Spherical	371.4/1/100	1
ZnSe@C	polyhedral	361/0.1/150	2
$Cu_{1.8}$ Se@C	decahedron	416/1/500	3
This Work ($AlCuSe_2$)	Spherical	686/0.1/200	
		351 / 2000 / 4	

Reference

(1) Yang, S.; He, M.; Deng, X.; Feng, Y.; Huang, X.; Wu, K.; Bai, C.; Ke, J.; Xiong, D. Wafer-like FeSe2-NiSe2/C nanosheets as efficient anode for high-performances lithium batteries. *Chemical Physics Letters* **2020**, *746*, 137274. DOI: https://doi.org/10.1016/j.cplett.2020.137274.
(2) Zeng, L.; Fang, Y.; Xu, L.; Zheng, C.; Yang, M.-Q.; He, J.; Xue, H.; Qian, Q.; Wei, M.; Chen, Q. Rational design of few-layer MoSe2 confined within ZnSe–C hollow porous spheres for high-performance lithium-ion and sodium-ion batteries. *Nanoscale* **2019**, *11* (14), 6766-6775, 10.1039/C9NR00146H. DOI: 10.1039/C9NR00146H.
(3) Xiao, J.; Liu, H.; Huang, J.; Lu, Y.; Zhang, L. Decahedron Cu1.8Se/C nano-composites derived from metal–organic framework Cu–BTC as anode materials for high performance lithium-ion batteries. *Applied Surface Science* **2020**, *526*, 146746. DOI: https://doi.org/10.1016/j.apsusc.2020.146746.
